# Approaches to discern if microbiome associations reflect causation in metabolic and immune disorders

**DOI:** 10.1080/19490976.2022.2107386

**Published:** 2022-08-08

**Authors:** Marijana Basic, Dominique Dardevet, Peter Michael Abuja, Silvia Bolsega, Stéphanie Bornes, Robert Caesar, Francesco Maria Calabrese, Massimo Collino, Maria De Angelis, Philippe Gérard, Miguel Gueimonde, François Leulier, Eva Untersmayr, Evelien Van Rymenant, Paul De Vos, Isabelle Savary-Auzeloux

**Affiliations:** a Institute for Laboratory Animal Science, Hannover Medical School, Hannover, Germany; b Human Nutrition Unit, UMR1019, University Clermont Auvergne, INRAE, Clermont-Ferrand, France; c Diagnostic & Research Centre of Molecular Biomedicine, Institute of Pathology, Medical University of Graz, Graz, Austria; d University Clermont Auvergne, Inrae, VetAgro Sup, Umrf, Aurillac, France; e The Wallenberg Laboratory, Department of Molecular and Clinical Medicine, Institute of Medicine, Sahlgrenska Academy, University of Gothenburg, Gothenburg, Sweden; f Department of Soil, Plant and Science, “Aldo Moro” University Bari, Bari, Italy; g Rita Levi-Montalcini Department of Neuroscience, University of Turin, Turin, Italy; h INRAE, AgroParisTech, Micalis Institute, Université Paris-Saclay, France; i Department of Microbiology and Biochemistry of Dairy Products, IPLA-CSIC;Villaviciosa, Spain; j Institut de Génomique Fonctionnelle de Lyon, Ecole Normale Supérieure de Lyon, UMR5242 CNRS, Université Claude Bernard-Lyon1, Lyon, France; k Institute of Pathophysiology and Allergy Research, Center of Pathophysiology, Infectiology and Immunology, Medical University of Vienna, Austria; l Flanders Research Institute for Agriculture, Fisheries and Food (Ilvo), Merelbeke, Belgium; m Immunoendocrinology, Division of Medical Biology, Department of Pathology and Medical Biology, University Medical Center Groningen; Groningen, Netherlands

**Keywords:** Gut microbiota, metabolism, immunity, *drosophila melanogaster*, *caenorhabditis elegans*, zebrafish, rodent, pig, human, causality

## Abstract

Our understanding of microorganisms residing within our gut and their roles in the host metabolism and immunity advanced greatly over the past 20 years. Currently, microbiome studies are shifting from association and correlation studies to studies demonstrating causality of identified microbiome signatures and identification of molecular mechanisms underlying these interactions. This transformation is crucial for the efficient translation into clinical application and development of targeted strategies to beneficially modulate the intestinal microbiota. As mechanistic studies are still quite challenging to perform in humans, the causal role of microbiota is frequently evaluated in animal models that need to be appropriately selected. Here, we provide a comprehensive overview on approaches that can be applied in addressing causality of host-microbe interactions in five major animal model organisms (*Caenorhabditis elegans, Drosophila melanogaster*, zebrafish, rodents, and pigs). We particularly focused on discussing methods available for studying the causality ranging from the usage of gut microbiota transfer, diverse models of metabolic and immune perturbations involving nutritional and chemical factors, gene modifications and surgically induced models, metabolite profiling up to culture-based approached. Furthermore, we addressed the impact of the gut morphology, physiology as well as diet on the microbiota composition in various models and resulting species specificities. Finally, we conclude this review with the discussion on models that can be applied to study the causal role of the gut microbiota in the context of metabolic syndrome and host immunity. We hope this review will facilitate important considerations for appropriate animal model selection.

## Introduction

The human gut harbors trillions of microbes. The recent change of the traditional view that gut microbiota effects are not only limited to fermentation of food but also influence metabolism and immune status. This has led to the realization that these microbes can be considered as an instrument for maintaining health.^1^ During recent years, it has been proven that microbes in the intestine are influenced by external factors such as diet, antibiotics and many other environmental factors that may affect the microbiota-host interactions in both positive and negative ways. This demonstrates the plasticity of the gut microbial community and shed new lights toward the manipulation of microbiota function and activity, useful in improving metabolic and immunological health, especially in disease prevention and treatment. However, to develop successful microbiome-based therapeutics, the field needs to concentrate on causation and mechanisms. This involves moving past descriptive microbiota and health parameter analyses to studies deciphering mechanistic interactions of how commensal microbes affect different health outcomes, as this is a crucial step in translating microbiome findings into clinics.

Establishing causality in humans is hindered mainly by high complexity of the human microbiome as well as immense genetic and lifestyle differences among populations and individuals. Consequently, the majority of human studies are still observational, or are not designed to prove causal relationships between microbiota changes and development of disease. Although adapted application of methods such as Mendelian randomization or machine-learning approaches are discussed as a mean of improving causality in human microbiome studies,^2,3^ most of our knowledge regarding microbial causality stems from model systems. Many experiments in animal models have demonstrated the proof of principle that interfering in host-microbe interactions can contribute to delay or prevent diseases. Also, animal models have been instrumental in understanding potential causal relations between microbiota changes and physiological-metabolic perturbations providing insight in potential mechanisms.^4,5^ Variables involved in health outcomes can be tightly controlled in animal models. This can be achieved through application of gnotobiotic animals, genetically manipulated animals, strict environmental controls, and the ability to sacrifice animals at the desired time-point in the study supporting the use of animal models to perform mechanistic analyses and establish causality in host-microbiota interactions. However, the translation of these observed animal results to humans remains complex and challenging. To improve this, factors influencing the healthy microbiome and microbiome reshaping during different disease stages need to be addressed and better understood. This can be accomplished by combined application of studies performed in both humans and model systems, which need to be appropriately selected. In the present manuscript, we summarize information on five major animal models (*Caenorhabditis elegans, Drosophila melanogaster*, zebrafish, rodents, and pigs) in which the role of microbiota in development of metabolic and immunological perturbations can be analyzed. The aim of this review is to discuss all important factors scientists need to take into account to make sure that their model, even if not perfect, will be able to address the research question with a maximum knowledge/awareness of the confounding factors such as gut physiology, diet, choice of model of pathology, and microbiota composition. This ultimately may lead to a more confident approach when causal relations between microbiota function and host-health or disease have to be inferred.

## Part A. Methods for studying the causal role of gut microbiota

### Microbiota changes – a chicken and egg question

The intestinal microbiota executes numerous beneficial functions for the host health. These include synthesis of essential vitamins or metabolites such as short chain fatty acids (SCFAs) (mainly acetate, propionate and butyrate), degradation of food components into nutrients, and regulation of metabolic and immune responses.^1,6^ Over the last 20 years, changes in intestinal microbiota composition or function have been associated with inflammatory, immune, metabolic, and behavioral disorders.^7–9^ However, most of these studies so far still demonstrate associations between microbiota alterations and host changes. In most instances, it is still unclear whether the observed changes are a cause or just a consequence of the disease progress. Therefore, a major and timely challenge is to infer causality from host and microbiome interactions. This will grant us to develop targeted strategies to prevent disease and modulate intestinal microbiota to the benefit of the host. To this end, the field is currently striving to decipher molecular mechanisms underlying host-microbe interactions and to gain insight in how this is related to host physiology and health status.

### Gut microbiota transfer as a mean of demonstrating causal relations

Gnotobiotic invertebrate and vertebrate animals or Fecal microbiota transfer in germ-free rodents are commonly used to establish causal relations between given a gut microbiota as a whole or its constituents and host phenotypes. It involves the colonization of ex-Germ Free (GF) invertebrate of vertebrate animals with given microbial culture or communities and the study of the resulting animal phenotypes^4^ or the administration of minimally manipulated microbial communities from the fecal or cecal matter of a donor (being an experimentally challenged animal of human patient) into a recipient GF mice to investigate the transfer of specific phenotypes of disease.^10–12^ Although this latter method represents a standard in the field there is much debate on whether the protocols applied allow a sound interpretation of the microbiota induced effects. In human-to-mice transfer studies, there is only the option to transfer human fecal matter, while in mice-to-mice transfer cecal microbiota is often preferred. This microbiota population contains all the microbiota needed for fermentation as the cecum is the primary site for generation of SCFAs. The microbiota can be transplanted into the same species (allogenic microbiota transfer) or into different species (xenogenic microbiota transfer). The predominantly used animal model for microbiota transfer is mice, but other species such as rats or pigs (mini pigs in general) can also be used as recipients.^13–15^ The microbiota transfer is mainly administered via intragastric inoculation.^10−12−16^ The preparation of donor inoculum for transfer varies from inoculation immediately after collection to administration of frozen samples with or without addition of cryoprotectant ^10−12^. Frequency of administration also varies from study to study ranging from single to multiple gavage cycles.^10,12,17,18^ All these variations might have impact on the outcome of a phenotype.

The microbiota recipients can be GF mice or conventionally raised mice with or without microbiota depletion by antibiotics^17,19^ (Table 1). While GF models, predominantly GF mouse models, are set as a benchmark for the studies of the microbiota impact, many researchers turn to microbiota-depleted models as a rapid, cheaper, and more accessible alternative. Complex microbiota of the recipients is reduced by removing a high proportion of endogenous taxa with broad-spectrum antibiotics. Studies showed that broad-spectrum antibiotic combinations such as ampicillin, vancomycin, neomycin, and metronidazole are more efficient than a single antibiotic to improve microbiota engraftment.^14,18^ However, usage of antibiotics cannot eliminate all intestinal microbes and can be associated with off-target drug effects, which needs to be considered when assessing the results. Recently, bowel cleansing with laxative-based approaches, such as polyethylene glycol (PEG), has been suggested as an alternative to GF or antibiotic-depleted models for microbiota transfer studies^17,18^ (Table 1). It is important to note that all of these approaches have their advantages and limitations that need to be considered when planning experiments (Suppl Table 1).

**Table 1.** Models of microbiota depletion: germ-free, antibiotic- and polyethylene glycol-induced including their specificities and research applications.

Model Feature	Germ-free	Antibiotic-induced microbiota depletion	Polyethylene glycol (PEG)-induced microbiota depletion
Derivation/ maintenance	Labor intensive and costly, specific equipment needed for maintenance	Inexpensive and accessible, no specific equipment needed for maintenance	
Availability	Only few strains commercially available, new strains need to be first re-derived germ-free	Usage of available complex microbiota-colonized models	
Treatment effect on the host	Physiological and anatomical special features, underdeveloped immune system	Drug off-target effects on host physiology and disease onset, primed immune system during neonatal period	Effects of significant microbial reduction on host physiology, primed immune system during neonatal period
Microbial composition/standardization	Fully known, absence of all living microorganisms, highly standardized	Unknown, presence of viruses, fungi, archaea, and antibiotic-resistant bacteria, facility-specific differences can be observed	Unknown, presence of residual microbes, facility-specific differences can be observed
Engraftment/ colonization stability	Long-term and intergenerational durability of engraftment	Negative effects of residual antibiotic and microbiota, potential loss of engraftment	Recovery of initial microbiota, loss of long-term engraftment stability
Research application/aim	Transfer of microbiota-induced phenotype (not known to be microbiota-dependent), effect of monocolonization or colonization with minimal microbial communities on host phenotype and physiology	Transfer of microbiota-induced phenotype (known to be microbiota-dependent), identification of bacteria relevant for different phenotypes, effects of microbiota disruption in different life stages	Transfer of microbiota-induced phenotype (known to be microbiota-dependent), effects of microbiota disruption in different life stages

Several human disorders such as obesity, inflammatory bowel disease, or malnourishment have been successfully transferred to mouse models by microbiota transfer.^20–22^ It is known from different xenogenic fecal transfer studies that the microbiota of the donor adapts to the recipient-microbiota composition during several weeks.^23,24^ This time lapse has proven to be efficient for the microbiota to induce changes in the mouse recipient and consequently resulted fundamental to unravel the mechanisms useful to determine whether specific immunological processes are microbiota dependent or not.^23,25^ Recent studies show that similar adaptations occur when transferring between mice strains and has still led to meaningful conclusions on which bacterial species are responsible for immune regulatory processes.^26^ Overall, these studies emphasize that microbiota humanized mice can reflect both the dysbiotic features of the microbial community and the disease phenotype, despite known limitations.^27^ This suggests that this model is still a useful tool to untangle disease mechanisms and identify disease- or health-relevant taxa. Nevertheless, using immunologically or metabolically humanized rodents or hosts with similar physiology such as pigs or primates will increase the possibility of identifying species involved in human-specific host-microbe interactions.^27^ However, there are and will be limitations as recently illustrated in a study involving 1700 transfers of human-to-GF mouse transfers, where less than half of the bacterial species identified in the human donors were able to colonize in GF mice.^28^ This can be attributed to several factors such as donor-to-donor variations in colonization efficiency due to individual donor characteristics that influence microbiota behavior such as differences in dietary habits, genetics, and lifestyle.^29^ In addition, some human species such as some *Firmicutes* and *Faecalibacterium spp*. are difficult to study in mice.^14,26^ A recent study also suggests that human microbiota colonize better in GF piglets than in GF mice, but this obviously needs confirmation in other studies and would not be as cost effective as mice studies.^30^

As environmental conditions are easier to master in smaller animal models, the use of non-mammalian model to investigate a simplified host-microbiome interaction is *de facto* easier to handle. In *Caenorhabditis elegans* or *Drosophila melanogaster*, for instance, GF animals can be fed with defined diets supplemented with pure culture of specific bacteria species or consortia that may differ from the microbial environment they usually encounter in the wild.^31^ For *C. elegans*, several studies have investigated the role of chosen specific bacterial strains (or consortia) – frequently lactic acid bacteria, on lifespan, fat metabolism, or metabolic pathways involved in energy metabolism regulation.^32,33^ The worm has also been extensively used in the field of host-pathogen interaction, triggering the regulation of innate immunity^34^ as well as a cascade of a wide variety of genes.^35^ Similarly, *Drosophila* has been extensively used as a model to study the phenomenology and the underlying molecular and cellular mechanisms of microbiota-diet interactions and their influence on host biology including postnatal development (ie juvenile growth and maturation), adult physiology (ie immune and metabolic functions and behavior) and aging.^36,37^

### And various models of metabolic or immune perturbations

Not only microbiota transfer studies but also exploring and studying influence of microbiota in animal models with diseases are used to unravel whether and how microbiota changes can cause disease. In order to find causal link, obesity, insulin resistance (IR), diabetes and cardiovascular diseases have been recently extensively inspected.^5^ The feasibility of such approaches relies on the characterization of microbiota communities responsible for disease developments as well as on microbiota deletion or correction with pre- and probiotics that may act to delay or prevent diseases. However, also immune disorders such as inflammatory bowel disease, allergies, and even autoimmune disorders such as type 1 diabetes are studied in animals.^38^ As observed in humans, these models generally reproduce one or several phenotypes of diseases and can be categorized in three groups depending on the way metabolic or immune disorders are generated: 1) homeostasis perturbations induced by environmental factors such as nutritional or chemically induced factors, 2) genetically modified polygenic or gene knock-out (KO) animals, or 3) surgically induced disorders. In many cases, these models have been set up without taking into account or even studying microbiota changes but they have the benefit that they are very well characterized and generally validated for translational research in humans.^5^ Importantly, confounding parameters such as animals’ diet, age, handling, environmental parameters^5^ need to be evaluated as potential interfering factors in causal relationships between microbiota changes and host responses. In parallel, the use of genetically modified (for genes involved in metabolic pathways or immune regulation) *Drosophila* or zebrafish lines can add highly valuable and novel concepts of mechanisms at stake in host-microbiota cross-talk and regulation of host genes by specific microbes.

### Microbial metabolites and their impact on host health: an indirect way to decipher the host-microbiota interaction

The intestinal microbiome is an integral part of the metabolism of nutrients and orally administered drugs. Consequently, the metabolome profile that results from nutrient processing by intestinal microbiota depends both on the nature of the consumed molecules and microbiota composition. These microbial metabolites are known to influence the severity and development of metabolic diseases, such as fatty liver disease, atherosclerosis, obesity, type 2 diabetes, and other manifestations of the metabolic syndrome (MetS). These microbial metabolites include SCFAs,^39^ indoles, secondary bile acids, biogenic amines, vitamins, ethanol, succinate, or pyruvate.^40^ These molecules have been linked to both positive and negative health outcomes depending on the context and disease in which they were studied.

Microbial metabolites affect both host physiology and microbiota composition as well as function. Bile acids for example are metabolized by the intestinal microbiota and are known to influence bile acids metabolism and hormonal activity.^41^ Also bile acids shape gut microbiota through influencing detergent activity and influence innate immunity (mainly microbiota derived-secondary bile acids for the later).^42,43^ SCFAs are known for their beneficial role on gut permeability, insulin sensitivity, and appetite-body weight regulation.^44^ This has been particularly investigated in rodents with supplementations or colic infusions of SCFAs^45^ but impact of microbial metabolites on host phenotype can also be studied in invertebrates. For instance, *C. elegans* is able to detect diverse metabolites (beneficial or detrimental) produced by broad classes of bacteria that can induce innate and learned behavioral responses but also modify longevity, revealing the influence of host-microbe interactions on phenotype of this invertebrate animal host.^46–48^ Similarly in *Drosophila*, a systematic work conducted on gnotobiotic animals bred on a large array of chemically defined diets has allowed to identify the full nutritional requirements of the GF host and how *Drosophila* commensals compensate for specific host auxotrophies and support their animal host nutrition and growth.^49^

### Culture-based approaches

To establish causality between microbial signatures and disease or health status of the host, robust experimental models with defined microbiota are required for validation of generated hypotheses. Some prefer the approach of defined culture-based microbial communities. This approach relies on isolation and characterization of intestinal microbiota members. In recent years, significant efforts have been made in this field resulting in representative strains available in pure culture. This however only applies to 35–65% of the species that can be detected by sequencing^50^ and still quite a number of microbial taxa are not taken into account because of inability to culture these microorganisms. For further development of the microbiome field, it is crucial that newly isolated and characterized commensal species are deposited in culture collections and in nucleotide (or taxonomic) databases making them available to the scientific community. Efforts are now being made to expand the collection of human, pig, and mouse gut bacterial isolates which are now the foundation of mechanistic studies.^51–53^ This approach facilitates studies on impact of single taxa on the host physiology, but also generation of defined beneficial microbial communities. Generation of models with simplified microbiomes can reduce influence of confounding effects of endogenous complex microbiota on host health effects and increase the experimental reproducibility. Assemblage of simplified microbiomes represent modular system in which intestinal isolates are combined together based on their function or scientific hypothesis to perform targeted mechanistic studies.^50–52^

## Part B. Animal models applicable for determining causality of microbiota changes and host phenotype

A very challenging assignment for every study addressing causal relationships is choosing an adequate model for studying a disease condition and carefully consider any parameters that might affect the gut microbiota. This requires appropriate knowledge of the characteristics of each animal model, its morphology, physiology, dietary habits, metabolism, microbiota composition but also genetics, behavior, and test environment (Suppl. Table 1 and Table 2).

**Table 2.** Gut and intestinal epithelium physiology. Comparative analysis between *C. elegans, Drosophila*, zebrafish, rodents, pigs and humans.

	*C. elegans* 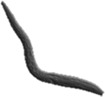	*Drosophila* 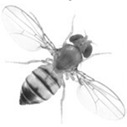	Zebrafish 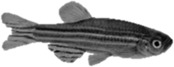	Rodents 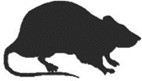	Pig 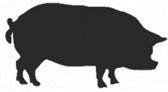	Human 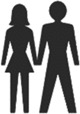
Gut Anatomy	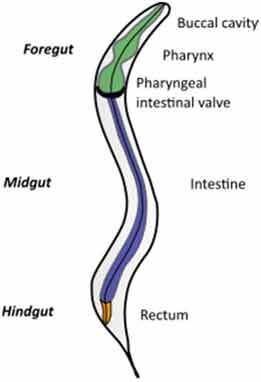	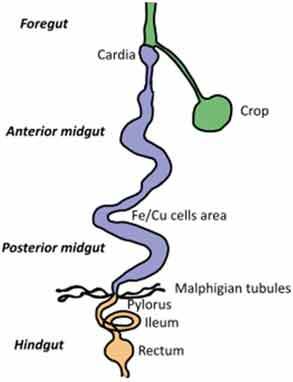	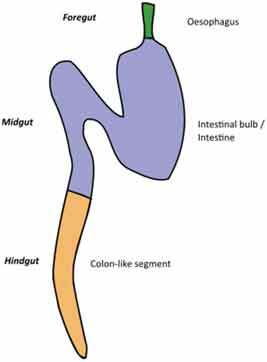	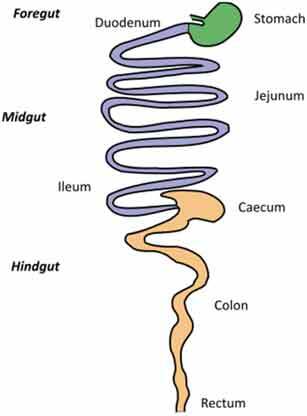	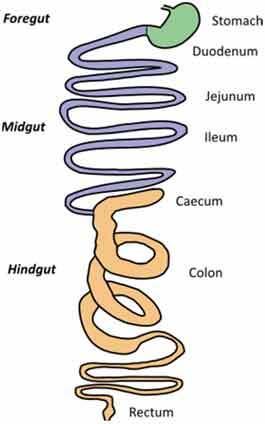	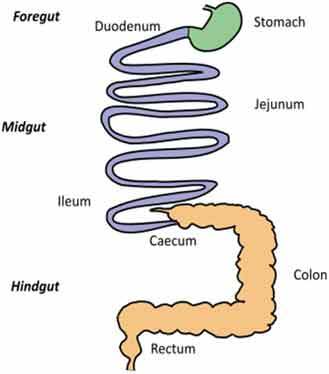
Gut physiology	Physiologically segmented gut/no clear analogies with human gut	Not morphologically segmented but three physiologically different gut segments Proximal foregut (oral cavity, esophagus, crop) Midgut (analogous to small intestine) Distal hundgut (analogous to colon)	Three different gut segments No stomach but anterior intestine Non glandular (pH never<7.5) Sections 1–5 analogous to small intestine (duodenum and jejunum like) Sections 6–7 analogous to colon	Different gut segments Partially non glandular stomach No gall bladder in rats, present in mice Small intestine (duodenum, jejunum ileum) Cecum Colon	Different gut segments Glandular stomach Small intestine (duodenum, jejunum ileum) No appendix and more Large cecum Spiral orientation of colon	Different gut segments Glandular stomach Small intestine (duodenum, jejunum ileum) Appendix Cecum not differentiated from colon Colon
Intestinal epithelium specificities	No crypts but microvillosities Simple layer of cells Glycoproteins to protect against pathogens	No crypts Enteroendocrine cells Enterocytes Reduced mucus layerbut chitinous matrix instead	No crypts (fingerlike protrusions) No organized lymphoid structures Goblet cells Enteroendocrinecells Enterocytes Presence of mucin	Crypts Organized lymphoid structures Paneth cells Goblet cells Enteroendocrinecells Enterocytes Smooth mucosal surface No plica circularis in small intestine, no haustrations in colon/cecum	Crypts similar to human Organized lymphoid structures Paneth cells Gobletcells Enteroendocrine cells Enterocytes Mucosal surface with folds Plica circularis in small intestine, haustrated colon/cecum	Crypts Organized lymphoid structures Paneth cells Goblet cells Enteroendocrine cells Enterocytes Mucosal surface with folds Plica circularis in small intestine, haustrated colon/cecum

*For gut anatomy pictures: foregut is in green, midgut is in purple and hindgut Is in orange*

### Gut morphology and physiology

Although similarities exist between species, digestive tract morphology, physiology as well as the amount and type of microbiota may vary (Table 2). Animals adapt to their environment and in particular to their food pattern.^54^ Omnivore species such as humans depend on food digestion and nutrient absorption in the foregut and midgut^54^ and on hindgut bacterial fermentation of food components that are not digestible by host enzymes. Anatomical differences of the hindgut exist between animal models used (Table 2). These differences need to be considered for translation of concepts to humans. In addition, physiological/biochemical discrepancies in the lumen environment (digestion rate, transit time, physical pressures, pH, osmolarity, enzymes, bile acids, metabolites) impact the metabolic fate of ingested nutrients and induce various selective pressure on microbiota present in the different segments of the intestine. If these concerns can be addressed partially in mammal models that present physiological similarities with humans, this is less the case in invertebrate models such as *C. elegans* or *D. melanogaster* where gut physiology, digestive processes, and nutritional habits are quite different from humans. Nevertheless, at cellular level, hydrolysis of lumen molecules by proteases, processes of metabolites absorption, lipids accumulation, endocytosis mechanisms and regulation of some metabolic pathways are partially preserved, and represent a complement, alternative and potentially more powerful option to investigate these mechanisms.^55,56^

In practice, mammals have been extensively used in digestion/absorption studies, but it should be pointed out that even if gut (including hindgut) physiology and metabolism are closer to humans, a critical review^57^ pointed out some discrepancies between mammals and humans. For instance, the small intestine of mice is lacking anatomical niches for mucus-associated bacteria that are present in other rodents and humans. For translational research, anatomically, the human large intestine has features similar to pig (omnivore) and the dog. In addition, there are major differences in the volume of the intestines between species. In primates, the colon represents about 50% of the total volume of the digestive tract while it only represents 20% in humans, implying a large difference in fermentation capacity and contribution of fermentation products to overall energy metabolism of the host.^58^ Indeed, Stevens *et al*.^59^ demonstrated a direct correlation between the fermentation end products contribution to overall host energy supply and size of the hindgut: 2% for dogs, 6–9% for humans, and 10–31% for pigs. Although anatomical differences can be major between species, many homologies are found such as the differentiated cell types present in the gut,^60^ and several biological and physiological homologies (Table 2).

At the immunity level, model species can be categorized in models with both an innate and adaptive immune system (mammals and to a certain extent zebrafish) and a category with only an innate immune system (invertebrates) (Table 3). Still, at the molecular level, some host pattern recognition receptors such as Toll-like, NOD-like, or C-type lectin receptors, involved in the innate immune system, are described both in mammals,^61^ zebrafish,^62^ and invertebrates.^63^ In this latter field, the utilization of invertebrates or fish can significantly add meaningful information on how host and gut microbiota communicate. Consequently, when choosing a model, it should again be emphasized that specific parameters and pitfalls should be carefully considered to make and appropriate choice to study host-microbiota interaction and impact on immunity.

**Table 3.** Metabolic (metabolic syndrome/diabetes) and immune specificities. Comparative analysis between *C. elegans, Drosophila*, zebrafish, rodents, pigs and humans.

	**C. elegans** 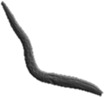	**Drosophila** 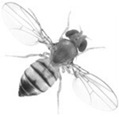	**Zebrafish** 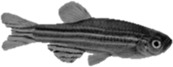	**Rodents** 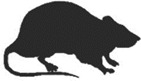	**Pig** 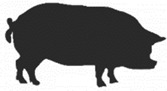	**Human** 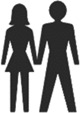
DIO/obesity/IR/diabetes model	DIO/diet restriction Accumulates fat/IR-like/IGF-1 like pathway Measurements: life expectancy Lipids accumulation (TG, lipid droplets staining) Mol. biol. methodologies ++ Insulin-like pathway No control of quantity ingested Control of type of food given	DIO/diet restriction Accumulates fat/IR-like/ insulin producing neurons /IGF-1 like pathway Measurements: life expectancy, body weight, body composition, hormonal profiles Lipids accumulation (TG, lipids staining) Mol. biol. methodologies +++ Insulin-like pathway/trehalose No control of quantity ingested Control of type of food given	DIO/diet restriction Accumulates fat/IR-like /β cells/IGF-1 like pathway Measurements: life expectancy, body weight, body composition, hormonal profiles Lipids accumulation (TG, lipids staining in larvae) Mol. biol. methodologies + Insulin pathway/glucose Complex control of intake Control of type of food given	DIO/diet restriction Accumulates fat/IR/β cells Many monogenic or polygenic models of diabetes or obesity Measurements: life expectancy, body weight, tissues weight, body composition, hormonal profiles Lipids accumulation in body and tissues (TG, histology) Mol. biol. methodologies ++ Insulin signaling /glucose, OGTT, ITT, HOMA-IRControl of intake Control of type of food given	DIO/diet restriction Accumulates fat/IR/β cells Some monogenic or polygenic models of diabetes or obesity Measurements: life expectancy, body weight, tissues weight, body composition, hormonal profiles Lipids accumulation in body and tissues (TG, histology), Mol. biol. methodologies, Insulin signaling /glucose, OGTT, ITT, HOMA-IR Control of intake Control of type of food given	DIO/diet restriction Accumulates fat/IR/β cells Measurements: body weight, body composition, hormonal profiles Lipids accumulation in body and tissues (TG, histology) Mol. biol. methodologies, insulin signaling /glucose, OGTT, ITT, HOMA-IR Assessment of intake (intake controlled in some controlled interventional studies)
Immunity	Innate/no adaptive immunity/lack of cell mediated immunity Main receptors/Innate immunity:TOL-1 (Toll homolog) No NLROther: CTLD, GPCRs	Innate/no adaptive immunity Main receptors/Innate immunity:9 Toll homologs, most pathogen defense relies on Toll-1, MyD88 No NLR Other: CTLD, PGRP, SR (CD36), GPCRs	Innate/late adaptive /cell-mediated immunity Main receptors/Innate immunity: TLR 1–5, 7–9, 14,18–22 NLRs Other: RLR, PGRP, GPCRs	Innate/adaptive/cell mediated immunity Main receptors/Innate immunity: TLR 1–9, 11–13, MyD88 NLRs (NOD1, NOD2) Other: CTLD, RLR, PGRP, SR (CD36), GPCRs	Innate/adaptive/cell mediated immunity Main receptors/Innate immunity: TLR 1–10, MyD88 NLRs (NOD1, NOD2) Other: CTLD, RLR, PGRP, SR (CD36), GPCRs	Innate/adaptive/cell mediated immunity Main receptors/Innate immunity: TLR 1–10, MyD88 NLRs (NOD1, NOD2) Other: CTLD, RLR, PGRP, SR (CD36), GPCRs

*Abbreviations: DIO: Diet induced obesity, IR: insulin resistance, IGF-1: Insulin growth factor −1, TG: triglyceride, mol. biol. molecular biology, OGTT: oral glucose tolerance test, ITT: insulin tolerance test, HOMA-IR: Homeostasis model assessment -insulin resistance, TLR: Toll like receptor, NLR: Nod like receptor, CTLD (C-type lectin receptors), RIG-like helicase receptors, PGRP: Peptidoglycan recognition proteins, SR: scavenger receptors, GPCRs: G protein receptors,*

### Diet

#### General considerations in mammals

In humans, it is widely accepted that microbiota composition and diversity are highly dependent on diet^64^ which can partially explain the variation in microbiota composition between and within individuals over time.^65^ Clear diet-correlated differences in microbiota composition have been demonstrated between vegetarians and meat-eating individuals^66^ or following interventional studies using pro- or prebiotics.^67^ At the same time, whole-community shotgun metagenomics sequencing applied to healthy subjects shows how, at the genomic level, sample phenotypic differences can be attributed to a small number of genomic changes and how the majority of genomic information is part of stable microbiome cores reflecting similar metabolic traits.^68^ Notwithstanding the need for an integrated approach with other omics data in deepening the metabolic traits (gene, transcript and protein catalogs), the microbiota adaptation to dietary habits can first of all be proven in a healthy status by studying changes in commensal species that can shift in favor of the host (commensalistic symbiosis) or can lead to positive outcomes for both the microorganisms and the host (mutualistic symbiosis).

The human microbiota-based sample clustering also applies to different species. Carnivores, omnivores, or herbivores have different microbiota composition but also contain clusters that are relatively conserved throughout species.^69^ It is not only the microbiota composition that is different but also microbiota functionality as the microorganisms produce different types of metabolic products such as SCFAs.^40^ Microbial ecosystems adapt to available nutrients, at both the diversity/composition and functional level. This is associated with a rapid adaptation of the enzymatic equipment of microbes^70^ and ability to synthesize microbial products such as SCFAs.

Although the timeframe required to induce shifts in microbiota composition is still subject of debate, it has been shown that shifts may happen in a matter of days or hours after change of a diet. Turnbaugh *et al*.^10^ have demonstrated a rapid transition in mice that presented an alteration in their microbiota composition and its gene expression profile within one day after switching from a high-plant carbohydrate diet to a high-fat, high-sugar diet. Also, circadian rhythms in microbiota composition have been observed both in humans and rodents, partially attributable to the fasting-feeding pattern.^71^ Longitudinal variation in the gut microbiota highlights the difficulty to properly assess microbiota composition, even within the same individual.

Lastly, feeding behavior is an underestimated but essential consideration when choosing an appropriate vertebrate model in translational research.^57^ In contrast to humans that consume food after several hours of fasting, mice and rats eat almost continuously during their period of activity, *i.e*. during the night.^72^ Because of this feeding pattern, food particles are constantly mixed with stomach fluid which results in a higher gastric pH (around 2.7–4.1) compared to humans. This is probably the reason why Lactobacilli are found in the upper part of the gut in mice while in humans only acid resistant bacteria like Streptococci, *Prevotella spp.*, and *Helicobacter pylori* are present.^73^ Additionally, coprophagia that is frequently observed in rodents, pigs, and rabbits can interfere with human translation since it is considered to be an important factor of microbiota modulation in coprophagous animals.^74^

#### Microbiota composition and species specificities: the case of mice

Microbiota composition of different animal species used to study host-microbiota interaction, diet effects, and/or in translational studies have been described previously.^4,57^ The fact that there are differences of variable extent in microbiota composition at different taxonomic levels, makes translation of data sometimes challenging. Detailed phylogenetic and metagenomic analyses showed that while many common genera are found in the human and murine intestine, they differ strongly in abundance. Around 80 microbial gut genera were reportedly shared between mouse and man, which was confirmed by a comparison of murine and human 16S rRNA datasets.^75^ However, a comparative survey of the phylogenetic composition of 16 human subjects and 3 often used mouse strains indicated that their microbiota is quantitatively very different.^76^ Additionally, laboratory circumstances influence microbiota composition. Factors such as inflammatory status, host genotype, diet, cage, and inter-individual effects and even the mouse-breeding facility influence microbiota composition.^57^ To address these issues, an extensive mouse microbiome catalog has been recently published.^77^ This catalog compares the human and mouse intestinal microbiota and shows considerable similarity at the genus level with a total of 60 genera detected in the mouse gut microbiome core, of which 25 were shared with the core genera in the human gut microbiome. However, when the mouse microbial genes were compared with that of human, only 4% were found to share considerable identity. Nevertheless, almost 80% of the annotated functions were common between mouse and human microbial datasets, indicating significant functional overlap. These observations support the argument that microbiota-transfer models are, for many bacterial species, appropriate for translational purposes.

#### Microbiota composition and species specificities: the case of invertebrates

In the lab, *C. elegans* are generally maintained on nematode growth medium and fed with Escherichia coli OP50 strain. The composition of the worm microbiome is rarely examined when they are growing in their natural environment (i.e. rotting fruits, compost, meaning a more complex diet). A comparative analysis (three studies) of microbiome from *C. elegans* raised in natural habitats showed that the worms harbor a core microbiome (i.e. nonrandom microbial colonization) of 14 microbial families.^78^ Hence, as for mammals, the concept of a core microbiome exists in *C. elegans* that results from both the selection of microbes during the establishment of a symbiosis and also on the microbes present in the specific environment of these animals, including food. These observations strongly suggest that worms can be a highly relevant model when it comes to study the understanding of the mechanisms of selection of microorganisms by the host, colonization processes, and host genes-microbiota interactions. This is specifically relevant in models harboring gut colonization with a microbiota composition limited to 10–20 different species but mastered to mimic core microbiota observed in the wild.^79^ As observed in vertebrates, the variable part of the microbiota is dependent on environmental factors.^80,81^ Three types^82^ of *C. elegans* microbiome has been identified relative to their dominant microbial taxa, with the largest group of *C. elegans* strains, harboring 28 strains with the dominance of *Ochrobactrum*. Still, it remains important to note that *C. elegans* is fed nearly exclusively on microorganisms. This means that microbes are used as nutrients supplier to the worm, even if they also colonize the worm’s gut as stated above. Consequently, an axenic *C. elegans*, when left axenic on a long-term basis, is food deprived.^83^ In this model, it should be kept in mind that decreasing the bacterial supply to the animals, that cannot be fully compensated by other nutrients, corresponds to food deprivation or strong food restriction that itself can have important consequences on metabolism regulation and is known to increase life span.^84^

In both the lab and the wild, *Drosophila*-associated microbes fall into two major bacteria phylotypes dominated by the Acetobacter and Lactobacilli species.^85^ They proliferate on the nutritional matrix and as such are frequently ingested by *Drosophila* adults or larvae. Depending on the diet, developmental stage, age and health status, the abundance and composition of the gut communities change and evolve, and vary from individual to individual.^86^ Their persistence in the entire intestinal tract is modulated by their ability to resist the physico-chemical constraints of this environment rather than intrinsic ability to reside in this niche.^56^ Indeed most *Drosophila*-associated bacterial strains do not reside in the *Drosophila* gut but are rather transiting through the intestine and constantly re-ingested. Yet, in two recent studies, stable colonization or the adult most anterior intestinal regions by strains of Acetobacter spp. and Lactobacillus spp isolated from wild flies has been reported suggesting that a seed microbiota may exist in *Drosophila*.^87,88^ Long-term persistence in the gut (ie, residency) is an important biological parameter to consider when studying microbial ecology, microbial dynamics in the host, microbiota vertical transmission and host ecological and evolutionary trajectories. However, many *Drosophila* associated strains (persisting or not, from wild flies or lab *Drosophila* cultures) show a marked functional impact on the physiology of their host. These observations establish that residency is not required for *Drosophila* commensal bacteria to shape their host’s physiology but is probably an important attribute that has shaped *Drosophila* microbiota transmission patterns over generations.

## Part C: Models of determining casual role of gut microbiota in metabolic syndrome

Rodents have been widely used in the field to identify the causative role of microbiota in energy handling, storage, and regulation of metabolic pathways. Microbiota transfer (allogenic or xenogenic) studies combined with interventional studies targeting specific microbiota functionalities have been developed to study MetS and obesity. This includes diet-induced obesity (DIO) models, genetically modified MetS animals, and animals with surgery-induced obesity (Table 3). These studies have demonstrated the co-evolution of specific microbiota phenotypes with obesity and MetS. However, other species are used as well such as minipigs because they are physiologically and metabolically closer to humans (Tables 1,2,3). These studies in vertebrates are complemented with invertebrate studies such as in *C. elegans* or *Drosophila* melanogaster to identify possible specific molecular mechanisms. In this latter case, genetically modified strains are commonly used as a powerful tool to highlight the role of specific genes in pathways regulations, but changes of diets are also tested, particularly in the case of *Drosophila* (High sucrose diet)^89^ and for *C. elegans* (High sugar – high lipid diet, starch supplemented diet).^90,91^ However, even if microbiota may be involved in energy homeostasis regulation in both *Drosophila* and rodents, it should be noted that underlying regulatory mechanisms may be very different as shown in axenic fruit flies that are capable to store more energy compared to their conventional counterparts,^92^ whereas GF rodents are lean and resistant to high-fat diets (HFD) relative to conventional rodents.^93^ Still, even if it remains necessary to be cautious in the translation of the results to humans, the use of *Drosophila* and *C. elegans* is expanding to screen probiotic bacteria that could be efficient in humans on specific immune-metabolic health outcomes and traits conserved in these models (longevity, specific metabolic pathways, lipids storage. ^94–96^

### Gnotobiotic models

Data from GF animals and from microbiota transfer from obese, IR, diabetic or steatotic individuals into GF animals suggest that the gut microbiota contributes to the development of metabolic phenotypes. Although this has led to novel insights, it is unknown which specific microorganisms determine host phenotype and obesity development. Recent experiments have combined animal models with defined microbiota compositions in combination with various diets, including obesogenic diets, to investigate the complex interactions between diet, microbiota and the consequence on metabolites and signaling molecules generated.^97^ Aside from rodents, other mammal models such as pigs, dogs, and *C. elegans* or *Drosophila* have also proven to be powerful tool in understanding the interactions between obesity-related disorders and microbial ecosystems. If for pigs and dogs the health outcomes studied are basically the same as the ones measured in rodents, life-span, overall lipids accumulation and genes expression/protein contents or metabolites are more targeted in the smaller models.

### Diet induced obesity models

Although nutritional factors have been implicated in DIO, it is still unclear whether a shift in microbiota composition or a shift in food supply is responsible for metabolic changes. DIO can be established in *Drosophila* to zebrafish, rodents, and pigs^98–101^ but because of similarities in food and nutritional habits, rodents and to a lesser extent pigs are used for translational research to human. Some studies in these models have shown the value of microbiota-targeted intervention or treatments with microbial metabolites such as SCFAs in search for microbiota dependent effects on obesity.^39^ In addition, by analyzing metabolic adaptations and signals such as gut peptides, metabolites and hormones, these models can contribute to new hypotheses on mechanisms of diet-induced obesity.

### Genetically induced metabolic syndrome

Many genetically modified (GM) rodents, invertebrates, and fish models have been developed to induce alteration in energy or nutrient handling.^5^ Physiological responses to an energy imbalance vary greatly between strains within the same species, with variable resistance to MetS.^5^ Depending on the model/strain used, the phenotype is generated on normal or DIO diet. Microbiota composition in GM rodent models such as ob/ob or db/db has also been shown to be changed and to correlate with glucose intolerance and severity of obesity.^102^ However, these GM models are not capable to fully mimic all metabolic perturbations occurring in MetS in humans and do not allow straightforward translation of the results to human MetS. In rodents, for instance, some models suffer from expedited beta cell dysfunction, others are rapidly prone to DIO or hepatic steatosis.^5^ This is particularly the case in monogenic and KO models whose phenotype highlights only specific, reproducible but fragmented views on mechanisms involved in metabolic adaptations to MetS. On the contrary, in polygenic models, variable degrees of obesity, IR, and steatosis were observed. This variability seems to represent the wider range of MetS phenotypes that is also observed in humans and might therefore be of value for translational research. For MetS, rodents but also invertebrate and fish models are extensively used as well as some pig models.^103^ In all these species, gut microbiota dysbiosis has been demonstrated in DIO.^100^

Despite the remaining complexities, these models aid in understanding how host genetics, including which genes, can influence microbiota composition. The study of co-evolution of microbiota and host genetics throughout life in these models could bring valuable data on how host genetics can drive microbiota composition and function.

### Rodent models of nonalcoholic fatty liver disease

Nonalcoholic fatty liver disease (NAFLD) is considered as the hepatic manifestation of MetS. It comprises a spectrum of diseases ranging from simple and usually nonprogressive steatosis to nonalcoholic steatohepatitis (NASH), fibrosis, and cirrhosis. Intestinal dysbiosis favors development and progression of metabolic liver disease and is characterized in adult patients by reduced abundance of Bacteroidetes and elevated Prevotella and *Prophyromonas spp*.^104^ together with reduced diversity of the microbiota. Since the pathomorphological characteristics of NASH and alcoholic steatohepatitis (ASH) are quite similar, permanent exposure to ethanol may be one of the determinants of disease development.^105^

Comparison of GF and complex microbiota colonized mice has first highlighted that lack of gut microbiota leads to excess amounts and accumulation of CAR-ligands such as bilirubin, bile acids, and steroid hormones leading to altered liver xenobiotic metabolism which could favor NAFLD development.^106^ Comparisons of GF and complex microbiota colonized mice further revealed that the commensal microbiota prevents fibrosis upon chronic liver injury in mice^107^ or determines the susceptibility to liver injury^108^ indicating that the severity and/or incidence of liver diseases may be modulated in GF mice. Henao-Mejia et al further established the causative role of the gut microbiota in liver disease development using mouse models deficient in the pro-inflammatory multi-protein complexes inflammasome.^109^ These inflammasome-deficient mice exhibited exacerbated NAFLD phenotypes on methionine choline-deficient or HFD. Strikingly, co-housing of wild-type mice with these inflammasome-deficient mice resulted in exacerbations of glucose intolerance and obesity, hepatic steatosis and liver inflammation in wild-type mice. This suggests that dysbiosis itself can induce NAFLD progression. By transferring gut microbiota from mice with or without NAFLD to GF mice, Le Roy et al. showed that the gut microbiota was responsible for NAFLD.^110^ More recently, the transfer of a high-fat-shaped microbiota to GF mice induced hepatic lipoprotein secretion and microvesicular steatosis.^111^ However, a recent study indicated that the contribution of the microbiome in NAFLD may be overarched by genetics of mice. This corroborates the observation that exchange of the microbiome between NAFLD susceptible and resistant mouse strains did not influence the development of steatosis in response to HFD.^112^ This, however, should not be interpreted as a suggestion that the microbiota does not influence the NAFLD.

Indeed, fecal microbiota transplants from human to mice have also been explored to decipher the causative role of the microbiome in NAFLD. Inoculation of feces from patients with NASH or from healthy persons in GF mice led to NASH phenotype in the NASH-microbiota recipient mice.^113^ A recent study further established that steatosis can be triggered in mice following human microbiota transplant from NAFLD patients confirming that the gut bacteria play a causative role in fatty liver development.^114^ Moreover, the gut microbiota from one genetically obese child with Prader–Willi syndrome induced liver steatosis in GF mice fed with a normal diet, indicating that the gut microbiota could promote the onset of liver steatosis in mice independently from diet and genetic factors.^115^

### Surgical procedures: the case of by-pass surgery

Recent data have emerged on the role of microbiota in the improvement of health status of obese patients after bariatric surgery.^116^ Because of this, microbiota composition and function in models of Roux-en-Y gastric bypass and sleeve gastrectomy models of obese/diabetic animals have been investigated.^117^ As for DIO, bariatric surgery targets both microbiota but also host appetite and capacities for nutrient digestion absorption. Consequently, the respective role of gut microbiota shift and decreased appetite as causes of efficacy of bariatric surgery’s on weight loss is regularly questioned.^117,118^

## Part D. Causal role of gut microbiota alteration in host immunity

The gut microbiota plays an essential role in the development and maintenance of a fully functional immune system in both invertebrates and vertebrates. Studies using GF mice reveal that the absence of commensal microbes is associated with underdeveloped lymphoid tissues, impairment in myelopoiesis, defective T and B cell functions, and low numbers of circulating CD4 + T cells and antibody production, all of which can be restored by *de novo* colonization leading to increase disease resistance.^119,120^ Transfer studies in GF mice have been instrumental in identifying key bacteria that support different immune cell populations such as T helper (Th), cytotoxic T (Tc) cells, or T regulatory (Treg) cells. *Akkermansia muciniphila* for example has been shown to be a bacterium that can lower incidence of both type 1 and 2 diabetes probably by supporting generation of Treg cells and lowering Tc cell activity that target insulin-producing cells.^121,122^ Another gut microorganism with Treg enhancing properties is the gram-positive clostridium *Faecalibacterium prausnitzii*. This organism belongs to the most abundant members of the gut microbiota and encompassed 2–5% of the total human microbiota.^123^ It is considered to be a major butyrate producing bacterium and its potential to attenuate disease has been shown in chemically induced mice colitis models.^124^ Monocolonization of GF animals with *Bacteroides fragilis* evokes the release of a bacterial polysaccharide able to direct the maturation of the developing immune system in mice leading to correction of systemic T-cell deficiencies and Th1/Th2 imbalances in lymphoid tissues.^125^ Even for unraveling the influence of *Bifidobacterium* species, mice models have been instrumental despite the fact that mice have a lower abundance in this bacterium than humans, and in some mouse strains even absence. *Bifidobacterium* is associated with a more regulatory immune profile when transferred to mice.^126,127^

Although fungi are clearly part of the microbiota, their roles in immune defense are less studied. Recent studies showed how the mucosa-associated fungus *Malassezia restricta* exacerbates colitis in mice^128^ and how a persistent *Candida* spp. colonization in the mouse gut exerts immunological effects at distant sites, such as the lung.^129^ Similarly, a gut-associated filamentous fungal *Talaromyces* species, isolated from wild *Aedes aegypti* mosquitoes, has been demonstrated to alter *Ae. aegypti* physiology in a way that facilitates pathogen infection.^130^

The essence of microbiota in keeping immunity active has also been documented in GF zebrafishes.^131^ As in mice, the absence of microbiota in zebrafish larvae is associated with several structural alterations, including immature patterns of brush border enzyme activity and glycoconjugate expression, and a paucity of enteroendocrine secretory cells.^132^ Furthermore, zebrafish gut microbiota has been demonstrated to impact the expression of over 200 genes in the zebrafish intestine, many of which have also been observed in mice and are involved in innate immunity, nutrient metabolism, and intestinal epithelial differentiation and renewal.^24^

While most recent mechanistic *in vivo* studies convincingly support the causal role of the commensal microbiome in driving immune activation in health and in disease, we need to consider that chronic inflammation conversely may shape microbial dysbiosis and functions of microbial communities in an extensive crosstalk between host microbiota and immunity. For instance, in mice the loss of Toll-like receptor 5 (TLR5), a component of the innate immune system that is expressed in the gut mucosa, produces alterations in the gut microbiota that induce low-grade inflammatory signaling.^133^ Similarly, deficiencies of antimicrobial peptide production, such as occur in NOD2 mutant mice, are primarily responsible for uncontrolled *B. vulgatus* colonization, reduction of microbial richness, and expansion of *Proteobacteria* to attenuate the main phyla *Bacteroidetes* and *Firmicutes*, potentially contributing to the onset of a severe inflammation.^134,135^ The role of innate immune system and recognition of pathogens by host receptors is a concept that is also extensively studied in *C. elegans* and *Drosophila* that also possess an innate immunity (Table 3). For instance, and even if receptors are different, some intracellular pathways involved in activation of innate immunity remain partially conserved over evolution (some TLR-activated pathways in particular – For review:^136–138^).

In addition to the impact of host-microbiota interactions on innate immune function, recent research also uncovered mechanisms governing mutualism between the microbiome and the adaptive immune system. Intestinal secretory IgA antibodies have been reported to shape gut microbial communities throughout selective coating of commensal bacteria which may promote modification in the bacterial gene expression, influencing their metabolic processes as well as their biogeography and survival within the gut.^139^

Transfer studies have, however, also revealed that some key immunological differences are not microbiota dependent. One of these is the gender-bias in immune responses which is more type-1 interferon skewed in females than in males. This was considered to be associated with gender dependent microbiota differences but transfer studies have shown^23^ that this was more dependent on inherited x-chromosomal- and sex-hormone-dependent differences in innate immunity in females that deletes typical male species such as *Alistipes, Rikenella*, and *Porphyromonadaceae*.

Despite these supportive findings for determining causative relationships between microbiota and host immunity some critical notes should be placed for the choice of mice models as principal differences in outcome may occur when comparing GF- and antibiotics-treated mice. For example, *Faecalibacterium spp*. a major Treg inducing species had a lower abundance in human microbiota GF C57BL/6 (B6) mice recipients than in mice in which the recipient microbiota was deleted by antibiotics.^14^

This further emphasizes the importance of a clear research strategy and associated choice for a recipient type and experimental model that match the aim of the study.

## Conclusion: The challenge to find an adequate model for mechanistic evaluation of host-microbiota interactions in the obesity/metabolic syndrome and immunity regulation fields

**Figure 1.**
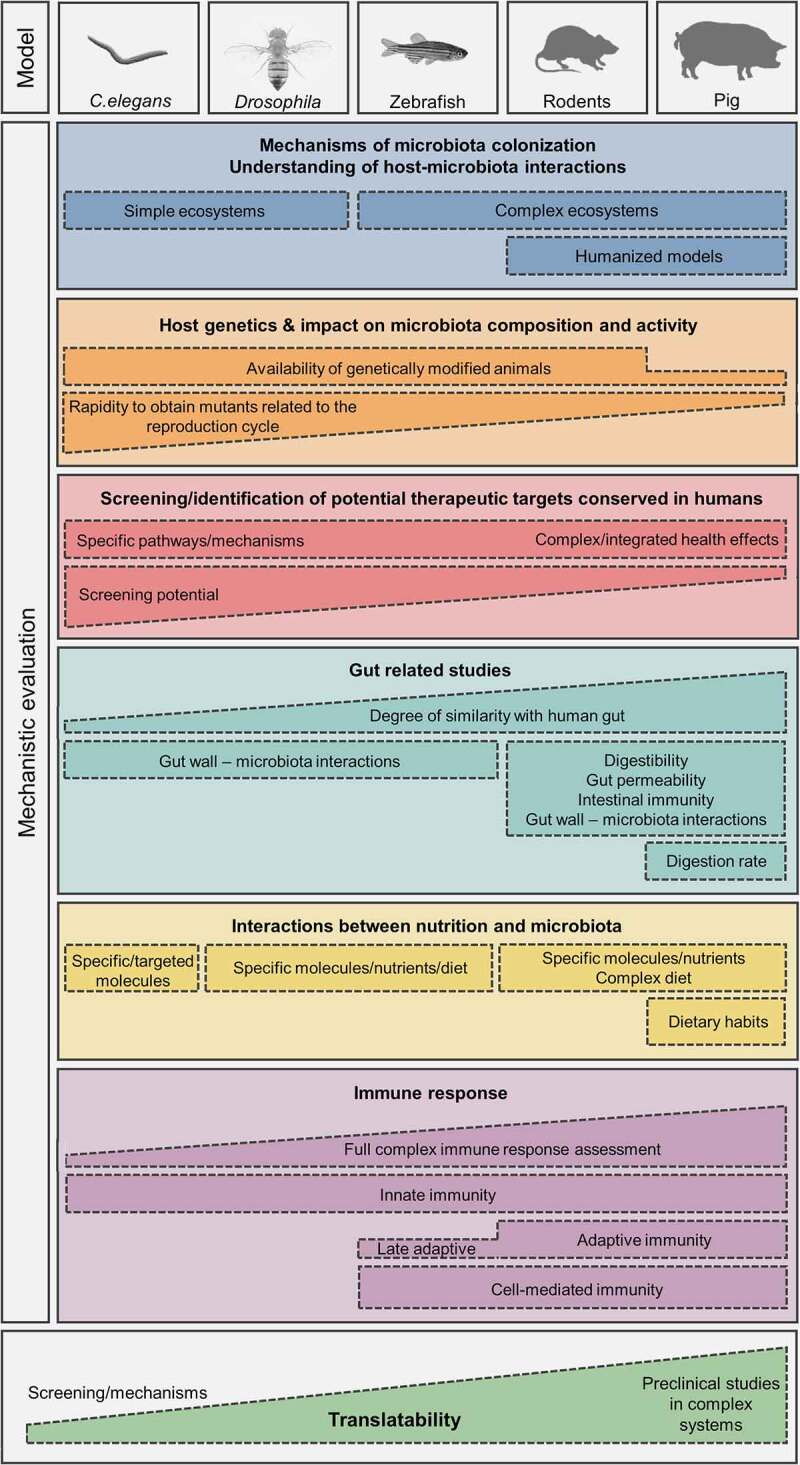
Decision making support for the choice of an appropriate animal model to study host-microbiota interaction mechanisms and causal role of microbiota in the development of MetS and immune response disruptions.

Each animal species that can be used to assess the complex and intertwined gut-microbiota-host health issue presents characteristics that should be considered as advantageous or detrimental depending on the scientific question addressed. Uncovering mechanisms that are underlying complex host-microbiota by resolving them to the level of specific metabolic or molecular pathway are vital to fully reveal the therapeutic potential of microbiota modulation. As the model species is more physiologically and microbially related to humans, the translatability of findings increases. On the contrary, when the role of a bacterial consortium needs to be evaluated, or more, when a very specific mechanism (conserved throughout evolution) is the target of the research, simpler models could be preferred. Although the straightforward and direct translation of findings from model systems is rather unfeasible, it can be increased by selection of appropriate models, by acknowledging and understanding confounding factors and by inferring appropriate conclusions. In Figure 1, the applicability of the main five models (*Caenorhabditis elegans, Drosophila melanogaster*, zebrafish, rodents, and pigs) for mechanistic evaluation of host microbiota-interaction in the fields of MetS and immune dysregulations is summarized. We hope that this review provides a comprehensive overview of important factors that scientist should be aware of when selecting a specific model to investigate the causal relation between microbiota changes and specific health outcomes.

Although our understanding of the role of gut microbes in host health and disease outcomes has advanced greatly over the past 20 years, there is still an immense knowledge gap that needs to be addressed in the future studies. This will facilitate the transition of microbiome research into the clinics and show the real power of microbiome-derived therapeutics. Currently, the interest and methodologies in the field of microbiota functionality are booming or being developed (integrative omics approaches, single-cell-based microbiota analyses, evaluation of the contribution of other microbiota members like fungi or phages, modeling of ecosystems assessing microbe-microbe interactions and consequences on metabolites production, microbial enzymes potentials obtained from metagenomics), which will in turn contribute to the better data interpretation gained from the model organisms. This better assessment of microbiota function will help to predict the host response that can be ultimately validated. Emerging area of interest is also the study of microbiota in different sites of the gut (such as mouth, jejunum, ileum, colon), but also other organs (lungs, gallbladder or liver) that are more rarely investigated, and the impact of these specific ecosystems on the host health. On the host side, the recent developments in the field of personalized therapies and nutrition have developed intensive research in the understanding of the determinants of variabilities within populations, including populations with MetS and immune disorders. Due to these recent conceptual and technical evolutions concerning host-microbiota interaction, no doubt that nutritional or drug strategies targeting microbiota will remain an active research topic. Lastly, and even if animal models remain essential in integrated physiology research, the burgeoning promising research using *in vitro* models (particularly organoids), but also fermenters are and will represent some interesting alternatives to address some questions related to host-microbiota interaction.

## Supplementary Material

Supplemental Material

## Data Availability

Data sharing is not applicable to this article as no new data were created or analysed in this study. All data and concepts described arise from the analysis of the literature detailed below (see Reference list).
