# Supplementary material for: Approaches to discern if microbiome associations reflect causation in metabolic and immune disorders
Source: Gut Microbes. 2022 Aug 8;14(1):2107386. doi: 10.1080/19490976.2022.2107386 (PMC9361767; doi:10.1080/19490976.2022.2107386)
Supplement: Supplemental Material [file KGMI_A_2107386_SM2749.docx]

|  | **C. elegans**  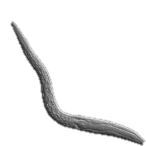 | 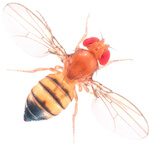**Drosophila** | **Zebrafish**  **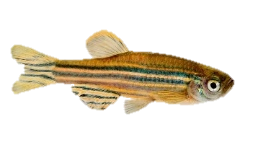** | **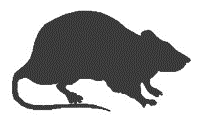Rodents** | **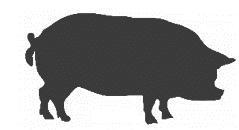Pig** | **Human**  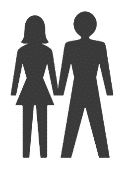 |
| --- | --- | --- | --- | --- | --- | --- |
| Model specificities vs. human | Metabolic pathways and biochemistry globally conserved  Short life cycle  Reproduction rate +++  Transparent  Terrestrial species | Metabolic pathways and biochemistry globally conserved  Short life cycle  Reproduction rate +++  Aerial and Terrestrial species | Metabolic pathways and biochemistry conserved  Short life cycle  Reproduction rate ++  Transparent when larvae  Aquatic species | Metabolic pathways, biochemistry and many gut physiology traits conserved  +/- short life cycle  Reproduction rate +  Terrestrial species | Metabolic pathways, biochemistry and major gut physiology traits conserved  Long life cycle  Reproduction rate +/-  Terrestrial species  Similar size to humans | Very long life cycle  Reproduction rate -  Terrestrial species |
| Nutritional specificities | Eats bacteria, microorganisms in general  Reference diet (*E. Coli* OP50)  No control of intake  Coprophagia | Eats bacteria  Nutritional requirements known / diets devoid of bacteria available /  Chemically defined diets/  Reference diets  No control of intake  Coprophagia | Nutritional requirements poorly known  No reference diet  No control of intake | Nutritional requirements known  Reference diets  Control of intake feasible  Coprophagia ++ | Nutritional requirements known  No reference diets  Control of intake feasible  Coprophagia + | Nutritional requirements known  No reference diets but nutritional habits |
| Model advantages vs. human | Axenic (easy +++) / gnotobiotic  Fully sequenced genome and available mol. biol. tools ++  Maintenance cost: low  Controlled environment +++  Genetic manipulation ++  Sampling at sacrifice  Repeated measures -  Reasonably simple microbiota, particularly when fed controlled bacterial diet / mechanisms of symbiosis easier to address  Ethical issues +/- | Axenic (easy +++) / gnotobiotic  Fully sequenced genome and available mol. biol. tools +++  Maintenance cost: low  Controlled environment +++  Genetic manipulation +++  Sampling at sacrifice  Repeated measures -  Reasonably simple microbiota / mechanisms of symbiosis easier to address  Ethical issues +/ - | Axenic (generally larvae) / gnotobiotic  Fully sequenced genome and available mol. biol. tools ++  Maintenance cost: low  Controlled environment +  Genetic manipulation ++  Sampling at sacrifice  Repeated measures -  Reasonably simple microbiota / mechanisms of symbiosis easier to address  Ethical issues + | Axenic (+/-easy) / gnotobiotic / humanized ++  Fully sequenced genome and available mol. biol. tools ++  Maintenance cost: average  Controlled environment +  Genetic manipulation ++  Sampling at sacrifice  Repeated measures +/-  Complex microbiota  Ethical issues ++ | Axenic (possible) / gnotobiotic / humanized +  Fully sequenced genome but mol. biol. tools +/-  Maintenance cost: high  Controlled environment +/-  Genetic manipulation +  Cannulation/catheters  Repeated measures +++  Complex microbiota  Ethical issues ++ | No axenic  Fully sequenced genome but not all tissues easily accessible  Clinical trials: high to very high  Controlled environment -  Genetic manipulation -  Cannulation/catheters  Repeated measures +  Complex microbiota  Clinical trials |

Abbreviations: mol. biol.- molecular biology

**Supplementary table 1: Physiological, nutritional and technical specificities of different model organisms.** **Comparative analysis between C. elegans, Drosophila, zebrafish, rodents, pigs and humans.**
